# Supplementary material for: Simple Model for Identifying Critical Regions in Atrial Fibrillation
Source: Phys Rev Lett. Author manuscript; Available in PMC 2015 Feb 25. (PMC4340556; doi:10.1103/PhysRevLett.114.028104)
Supplement: Supplementary Materials [file NIHMS61928-supplement-Supplementary_Materials.pdf]

1 Supplementary Materials

Risk of AF

We identify the risk of developing atrial fibrillation in a model with the probability, P_{risk}, that the $L \times L$ system has at least one portion of the reentrant circuit as displayed in Fig. 2(**e**). In our simple model, we can calculate P_{risk} analytically. The probability that a transverse coupling exists is given by ν. The probability that a cell has at least one transverse coupling, p_ν, is the complement of having no transverse couplings, that is,

$$p_\nu = 1 - (1-\nu)^2 \,. \tag{1}$$

Given an arbitrary dysfunctional cell i, let ℓ_i denote the distance (measured in number of links) from that cell to its first neighbour to the right that has at least one transverse coupling. Hence, the probability that $\ell_i < \ell_{\text{min}}$

$$P\left(\ell_i < \ell_{\text{min}}\right) = \sum_{\ell_i=0}^{\ell_{\text{min}}-1} (1-p_\nu)^{\ell_i} p_\nu = 1 - (1-p_\nu)^{\ell_{\text{min}}} = 1 - (1-\nu)^{2\ell_{\text{min}}} \,, \tag{2}$$

that is, the complement satisfies

$$P(l \geq \ell_{\text{min}}) = (1-\nu)^{2\ell_{\text{min}}}. \tag{3}$$

A micro re-entry circuit may not form if the path length of the potential re-entry circuit is less than the refractory period. That happens when $\ell_{\text{min}} = \tau/2$ (for even τ) and we find that

$$P\left(l_i < \frac{\tau}{2}\right) = 1 - (1-\nu)^{\tau} \,. \tag{4}$$

The average number of dysfunctional cells in a system is given by δL^2. P_{risk} is the complement of all dysfunctional cells having $l_i < \frac{\tau}{2}$, that is,

$$P_{\text{risk}} = 1 - \prod_{\substack{\text{dysfct.}\\ \text{cells}}} P\left(\ell_i < \ell_{\text{min}}\right) = 1 - \left[1 - (1-\nu)^{\tau}\right]^{\delta L^2} \,. \tag{5}$$

In the calculation above, we have treated all sites equally, that is, we have ignored boundary effects. We will now redo the calculation rigorously in a

finite system. The finiteness of the system implies that for a given dysfunctional cell i, there is a maximum distance $L-i$ to its first neighbour to the right that has at least one transverse couplings. Hence

$$\begin{aligned} P\left(\ell_i \geq \ell_{\text{min}}\right) &= \begin{cases} \sum_{\ell_i=\ell_{\text{min}}}^{L-1} (1-p_\nu)^{\ell_i} p_\nu & \text{for } \ell_{\text{min}} \leq L-i \\ 0 & \text{for } \ell_{\text{min}} > L-i \end{cases} \\ &= \begin{cases} (1-p_\nu)^{\ell_{\text{min}}} - (1-p_\nu)^{L-i+1} & \text{for } \ell_{\text{min}} \leq L-i \\ 0 & \text{for } \ell_{\text{min}} > L-i, \end{cases} \end{aligned} \tag{6}$$

and we find that

$$P\left(\ell_i < \ell_{\text{min}}\right) = \begin{cases} 1-(1-\nu)^{2\ell_{\text{min}}} - (1-\nu)^{2(L-i+1)} & \text{for } \ell_{\text{min}} \leq L-i \\ 1 & \text{for } \ell_{\text{min}} > L-i. \end{cases} \tag{7}$$

A reentrant circuit may not form if the path length of the circuit is less than the refractory period. That happens when $\ell_{\text{min}} = \tau/2$. The probability that all dysfunctional cells have $\ell_i < \frac{\tau}{2}$ is given by $\prod_{\substack{\text{dysfct.}\\ \text{cells}}} P\left(\ell_i < \tau/2\right)$. Note, however, that only dysfunctional cells with with $i = 1, \ldots, L-\tau/2$ contribute with a factor less than one. For each $i = 1, \ldots, L$, the average number of dysfuncitonal cells is δL. Hence, the risk that a system has at least one critical region is given by

$$\begin{aligned} P_{\text{risk}} &= 1 - \prod_{\substack{\text{dysfct.}\\ \text{cells}}} P\left(\ell_i < \tau/2\right) \\ &= 1 - \prod_{i=1}^{L-\tau/2} \left[1-(1-\nu)^{\tau} - (1-\nu)^{2(L-i+1)}\right]^{\delta L}. \end{aligned} \tag{8}$$

Note that for $\nu = 1$ and $\nu = 0$, $P_{\text{risk}} = 0$ for all values of $\delta L \neq 0$ and that $P_{\text{risk}} = 0$ when no dysfunctional cells are present, $\delta = 0$. It is also interesting to note that for $0 < \nu < 1$,

$$\lim_{L\to\infty} P_{\text{risk}} = 1, \tag{9}$$

that is, the quite abrupt transition from normal heart rhythm to fibrilatory behaviour seen when the fraction of transverse connections decreases below a critical value is technically an effect of the system having finite size. Un-

usually, though, as we will prove below, this finite-size effect disappear extremely slowly and hence it is physically relevant for all system sizes and is even present for systems with an area the size of the Earth.

Estimating the threshold for fraction of transverse connections ν

We define the threshold for the fraction of transverse connections as the value of $\nu^\star$ where the slope of the graph of P_{risk} vs. ν is steepest. For simplicity, we consider the result where boundary effects are ignored, that is,

$$P_{\text{risk}} = 1 - \left[1 - (1-\nu)^\tau\right]^{\delta L^2} . \tag{10}$$

We are searching for the non-trivial $\nu^\star$ that solves $\frac{\partial^2 P_{\text{risk}}}{\partial \nu^2} = 0$ We find that

$$\frac{\partial P_{\text{risk}}}{\partial \nu} = -\delta L^2 \left[1 - (1-\nu)^\tau\right]^{\delta L^2 - 1} \tau \left(1-\nu\right)^{\tau - 1} . \tag{11}$$

Hence, $\frac{\partial P_{\text{risk}}}{\partial \nu} < 0$ for $0 < \nu < 1$ implying that P_{risk} is a monotonic decreasing function of ν. We find that

$$\frac{\partial^2 P_{\text{risk}}}{\partial \nu^2} = -\delta L^2 \left[1-(1-\nu)^\tau\right]^{\delta L^2-2} \tau \left(1-\nu\right)^{\tau-2} \left[\left(\delta L^2-1\right) \tau \left(1-\nu\right)^\tau - \left[1-(1-\nu)^\tau\right] (\tau-1)\right] . \tag{12}$$

Therefore,

$$\frac{\partial^2 P_{\text{risk}}}{\partial \nu^2} = 0 \Leftrightarrow \nu = \begin{cases} 0 \\ 1 \end{cases} \tag{13}$$

or that $\nu^\star$ satisfies the equation

$$\left(\delta L^2 - 1\right) \tau \left(1-\nu^\star\right)^\tau - (\tau - 1) + \left(1-\nu^\star\right)^\tau (\tau - 1) = 0. \tag{14}$$

We solve this equation with respect to $1-\nu^\star$:

$$1 - \nu^\star = \left(\frac{\tau - 1}{\delta L^2 \tau - 1}\right)^{1/\tau} \approx (\delta L^2)^{-1/\tau}, \tag{15}$$

because to a good approximation, $\tau \gg 1$ and $\delta L^2 \tau \gg 1$. Setting $\delta = 0.05, \tau = 50$ and $L = 200$, we estimate the threshold $\nu^\star$ where P_{risk} picks up quite abruptly to be at

$$1 - \nu^\star \approx 0.86 \Leftrightarrow \nu^\star \approx 0.14. \tag{16}$$

It is interesting to note that the threshold approaches 1 like $L^{-2/\tau}$, that is, extremely slowly. For example, the threshold reaches $\nu^\star = 0.63$ for $L = 5 \times 10^{11}$, corresponding to an atria with an area the size of the Earth. Hence, although the transition is technically a finite-size effect, it is relevant for all system sizes and only disappears when $L = \infty$.

3D Sheet

We now calculate P_{risk} for multiple sheets. Consider w parallel sheets of size $L \times L$. Each sheet has periodic boundary conditions transversally and open boundary conditions longitudinally. This results in a cylindrical topology with cylinder thickness w. The probability that a cell has at least one transverse coupling, p_ν, is the complement of having no transverse couplings, that is,

$$p_\nu = 1 - (1-\nu)^n \,, \tag{17}$$

where $n = 3$ if a cell is at the surface and $n = 4$ if the cell is within the bulk of the tissue.

The probability that an arbitrary dysfunctional cell, i, is a distance $\ell_i < \ell_{\text{min}}$ becomes:

$$P\left(\ell_i < \ell_{\text{min}}\right) = 1 - (1-p_\nu)^{n\ell_{\text{min}}} \tag{18}$$

So the probability that all dysfunctional cells (on average $\delta w(L-2)^2$) have $\ell_i < \ell_{\text{min}} = \frac{\tau}{2}$ is

$$P_{\text{risk}} = 1 - \prod_{\substack{\text{dysfct.}\\ \text{cells}}} P\left(\ell_i < \frac{\tau}{2}\right) = 1 - \left[1-(1-\nu)^{\frac{3}{2}\tau}\right]^{2\delta L^2}\left[1-(1-\nu)^{2\tau}\right]^{(w-2)\delta L^2} \tag{19}$$

The curve still shows a sudden transition (see supplementary Fig. 1). We show this for $\delta = 0.05, \tau = 50, w = 7$ and $L = 200$.

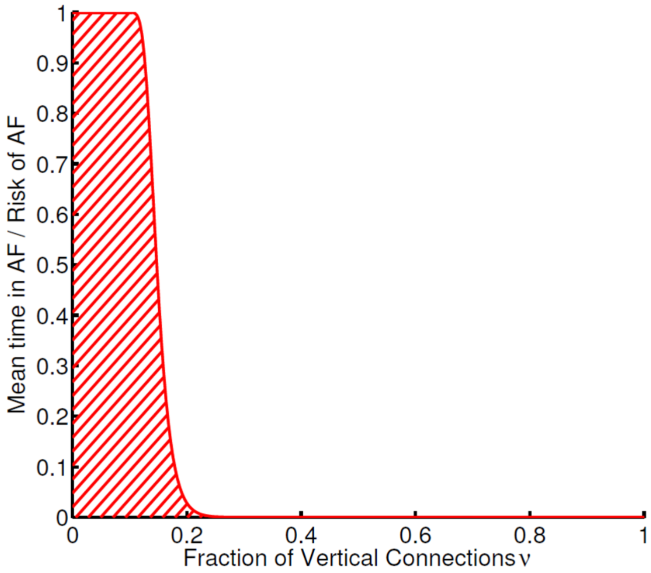

Figure 1: The probability that a three dimensional substrate, that is, a cylinder with finite thickness, develops AF, $P_{\rm risk}$. A sudden transition as the transversal coupling is varied remains.

Additional Figures

The figures shown in the manuscript were enlarged subsections of the full system size. For completeness we include the images for the full system size.

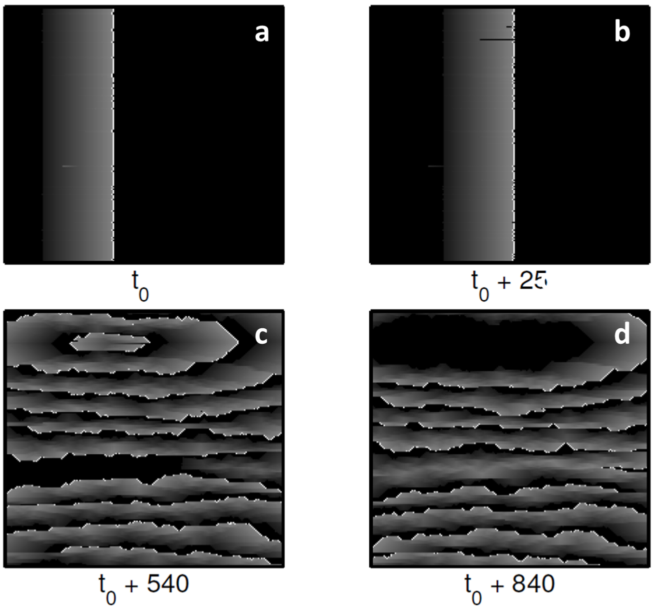

Figure 2: The initiation and termination of a re-entry circuit. This is the full 200×200 system of the sub-section show in Fig. 2 of the manuscript.

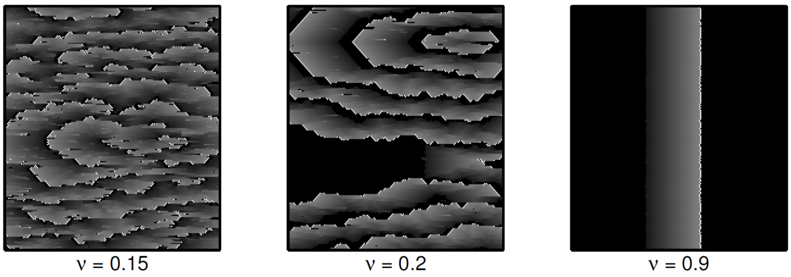

Figure 3: Complexity of wave behaviour at different levels of vertical coupling. This is the full 200×200 system of the sub-section show in Fig. 3 of the manuscript.

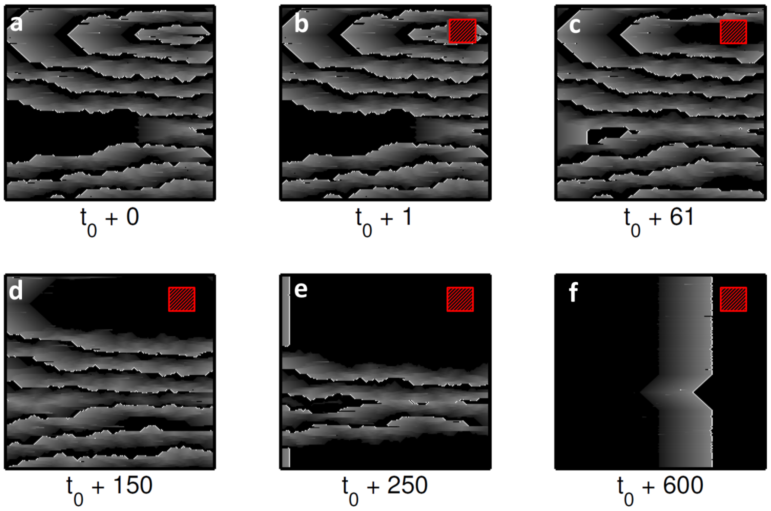

Figure 4: A region of tissue is made unexcitable simulating ablation. This is the full 200×200 system of the sub-section show in Fig. 4 of the manuscript.
